# Case Report: Spontaneous Intramural Hematoma of the Colon Secondary to Low Molecular Weight Heparin Therapy

**DOI:** 10.3389/fphar.2021.598661

**Published:** 2021-05-14

**Authors:** Ye Zhu, Chao Wang, Chao Xu, Jia Liu

**Affiliations:** ^1^ Clinical Medical College, Yangzhou University, Yangzhou, China; ^2^ Department of Biostatistics and Epidemiology, University of Oklahoma Health Science Center, Oklahoma City, OK, United States

**Keywords:** anticoagulation, bowel, hematoma, low molecular weight heparin, case report

## Abstract

**Background:** Hematoma of the colon is a rare hemorrhagic complication that affects patients accepting low molecular weight heparin (LMWH) therapy. Only scarce cases of colon hematoma have been reported, usually in children or patients accepting warfarin therapy.

**Case summary:** A 76-year-old Chinese man was diagnosed with atrial fibrillation and heart failure, with cardiac function NYHA grade III on March 21, 2018. This patient was given LMWH for anticoagulation therapy and developed a colon hematoma on the third day of hospitalization. Abdominal computed tomography (CT) showed the thickening of areas of the colon up to 110 mm × 78 mm in thickness, which was a symptom of colon hematoma. The patient underwent conservative treatment successfully. On March 27, the patient’s abdominal pain was alleviated, and a CT scan showed that the intestinal hematoma was absorbed.

**Conclusions:** The most frequent minor bleeding events of LMWH anticoagulation are hemorrhage and subcutaneous hematoma. This case demonstrated that bowel hematoma despite its low incidence should be considered as an ADR of LMWH therapy, especially among patients who present with gastrointestinal symptoms.

## Introduction

Low molecular weight heparin (LMWH) has become the preferred agent for the prophylaxis and treatment of thrombosis disease in patients as it has been shown to be safe and effective. It is also used as a bridging treatment of atrial fibrillation (AF) and then was switched to treatment with oral anticoagulants (Xia et al., 2018). As with any other anticoagulants, the main complication of LMWH therapy is bleeding. Most spontaneous gastrointestinal tract hematomas are caused by blunt abdominal trauma, which can also be secondary to anticoagulation therapy. Other risk factors for spontaneous gastrointestinal tract hematoma involve an endoscopic examination, coagulation disorder, and hemorrhagic disease (Zammit et al., 2013). Cases of spontaneous bowel hematoma associated with subcutaneous LMWH injection have been reported, while colon hematoma cases are very rare, usually in children or patients undergoing warfarin therapy (Chung, 2016; Choi et al., 2018). We herein present a novel case of spontaneous intramural hematoma of the colon associated with subcutaneous LMWH therapy.

## Case Presentation

### Chief Complaints

A 76-year-old Chinese man complained of acutely worsening abdominal pain after treatment with 4,000 anti-Xa U of LMWH, q12h (low molecular weight heparin calcium injection, 0.4 ml/4000IU) as an anticoagulant for 3 days.

### History of Present Illness

The patient was admitted to our hospital because of palpitation and shortness of breath for three days, and diagnosed with atrial fibrillation and heart failure, with cardiac function NYHA grade III at Northern Jiangsu People’s Hospital on March 21, 2018. He had a CHA_2_DS_2_-VASc score of 4, which indicated a high risk of stroke. His body mass index was 21.66 kg/m^2^, and renal function was normal. Therefore, he was given furosemide and spironolactone for diuretic therapy, valsartan capsules for antihypertensive treatment, and LMWH for anticoagulation therapy.

### History of Past Illness

The patient had a prior history of hypertension well controlled by treatment with angiotensin-converting enzyme inhibitors.

### Personal and Family History

The patient had no specific underlying disease. He had no family history of other significant diseases.

### Physical Examination Upon Admission

Physical examination showed abdomen tenderness and no signs of peritoneal irritation.

### Laboratory Examinations

On March 21, laboratory results showed that N-terminal pro–B-type natriuretic peptide was 1590 pg/ml, international normalized ratio was 1.14, activated partial thromboplastin time (APTT) was 38.20 s, fibrinogen was 1.67 g/L, hemoglobin was 113 g/L, red blood cell count was 3.34 × 10^12^/L, creatinine was 83 μmol/L, alanine transferase was (ALT) 39.0 U/L, aspartate aminotransferase (AST) was 36.0 U/L, gamma-glutamyltransferase (GGT) was 115.0 U/L, and platelet cell count was 183 × 10^9^/L. After anticoagulation therapy for 3 days, an emergency laboratory test showed that the red blood cell count was 2.56 × 10^12^/L, and hemoglobin and platelet counts decreased to 82 g/L and 102 × 10^9^/L, respectively. Furthermore, the coagulation function test demonstrated a prolonged APTT of 49.50 s. There was no bleeding per rectum, and his fecal occult blood test was negative. Considering the patient’s symptoms of abdominal pain and the rapid drop in hemoglobin and red blood cell count, there was clinical suspicion for retroperitoneal or gastrointestinal hemorrhage.

### Imaging Examinations

**FIGURE1:**
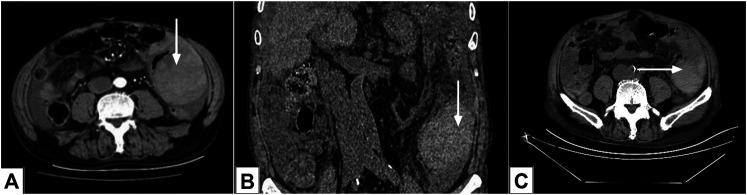
Abdominal computed tomography images. **(A, B)** Contrast-enhanced computed tomography (CT) images showing a hematoma of the colon (arrows) on March 23, 2018; C: CT image revealed that the colon hematoma had been absorbed (arrow) on March 27, 2018.

Abdominal computed tomography (CT) showed the thickening areas of the colon up to 110 mm × 78 mm in thickness, which was a symptom of hematoma of the colon (Figures 1A,B).

## Final Diagnosis

Hematoma of the colon took place after a subcutaneous LMWH injection. The patient had no medical history of hemorrhagic diseases, trauma, and any other anticoagulation therapy; we suspected that it was an adverse drug reaction (ADR) of LMWH. The assessment of ADR was evaluated *via* the Naranjo probability scale (Naranjo et al., 1981), which helps to identify the causal relation between an ADR and a drug based on the validated clinical questionnaire set by domain experts. The Naranjo scale consists of 10 questions which are administered for each patient’s clinical record. The Naranjo scale assigns a causality score, which is the sum of the scores of all Naranjo questions, that classifies the case into one of four causality types: doubtful (≤0), possible (1–4), probable (5–8), and definite (≥9). This patient had a Naranjo probability score of 8, which indicated that LMWH was a probable cause of this bleeding event. All the above examinations revealed a final diagnosis of hematoma of the colon, a rare ADR caused by LMWH therapy.

## Treatment

The patient accepted conservative management with bowel rest and intravenous fluids. His anticoagulation therapy of subcutaneous LMWH injection was discontinued immediately. Phloroglucinol injection (40 mg) as a musculotropic antispasmodic drug was prescribed to relieve abdominal pain. Tranexamic acid as a procoagulant was used to reduce the risk of bleeding, and omeprazole was prescribed to prevent gastrointestinal mucosal injury.

## Outcome and Follow-Up

On March 27th, the patient’s abdominal pain was relieved, and a CT scan showed that intestinal hematoma was absorbed (Figure 1C). The patient was discharged from hospital 2 weeks later.

## Discussion

Spontaneous bowel intramural hematoma is a rare complication under anticoagulant therapy. Warfarin is the most common cause of spontaneous intramural small-bowel hematoma in adults. The incidence of bowel hematoma was reported to be 1/2500 per year in patients receiving warfarin, and the incidence is relatively higher in males (Bettler et al., 1983). Limmer and Clement (2017) reported a case of successful conservative treatment of bowel hematoma caused by overdose anticoagulation with warfarin in a 71-year-old man. Shaw et al. (2005) reported one case of small-bowel hematoma in a child who received therapeutic doses of LMWH because of deep venous thrombosis. Approximately 85% of spontaneous intestinal intramural hematomas in patients with anticoagulant therapy occur in the small bowel (Xiao et al., 2015); however, the incidence of spontaneous hematoma is extremely rare in the colon. Thus, we herein present a novel case of spontaneous intramural hematoma of the colon associated with subcutaneous LMWH therapy.

Clinical presentation of bowel hematoma can vary from mild abdominal pain to intestinal obstruction or an acute abdomen. Nausea and vomiting are found in half of the cases and are related to intestinal obstruction. The average time from the occurrence of symptoms until medical attendance is 2.5 days (Sorbello et al., 2007). The diagnosis of bowel hematoma requires imaging data. Abdominal CT is currently the preferred imaging method for intestinal hematoma. Some people suggested that non-contrast CT should be performed for oral and intravenous contrast medium application, as contrast-enhanced CT alone may mask the presence of intramural hemorrhage. Most bowel hematomas can be treated conservatively, including discontinuing or reversing the anticoagulation and alleviating abdominal pain caused by intestinal obstruction. Surgery is indicated for complications or persistence of bowel hematoma (Zammit et al., 2013). Our patient was 76 years old, with a body mass index of 21.66. He had normal coagulation, liver, and kidney function at admission, and had no medical history of hemorrhagic diseases. Abdominal pain occurred 3 days after subcutaneous injection of LMWH, and abdominal CT scan indicated an intramural hematoma of the colon. This colon hematoma ADR is not mentioned in the official product information of LMWH.

LMWH has become the preferred agent for the prophylaxis and treatment of thrombosis disease. Compared with heparin, it has been shown to be safe and effective, with reduced incidence of heparin-induced thrombocytopenia (HIT) complication. LMWH molecular weight around 5000 Da is considerably variable in the chemical structure and has anti-factor Xa and anti-factor IIa activities (Hao et al., 2019). LMWH has a lower anti-factor IIa activity and a relatively higher anti-factor Xa activity. Subcutaneous LMWH injection is absorbed completely, with a half-life period of 3–5 h. While routine monitoring of coagulation parameters is not usually necessary for LMWH, certain populations (including pregnant patients, children, obese patients, and patients with renal impairment) may benefit from the monitoring of anti-factor Xa activity to help guide drug therapy (Levine et al., 2004; Sunseri et al., 2018). The main risk of LMWH, as with any anticoagulation agent, is bleeding. Hemorrhagic events that are reported usually include subcutaneous hematoma, hematuria, hemorrhinia, and gastrointestinal and retroperitoneal hemorrhage, while bowel hematoma rarely occurs. One of the mechanisms leading to bowel intramural hematoma might be the rapid decompression of splanchnic circulation due to decreased abdominal pressure, causing the bowel to rupture and bleed while he was on therapeutic anticoagulation therapy. There were limitations in our case; it was unclear whether LMWH dosage was a factor in the hemorrhage as the anti-Xa level was not monitored, and some other features such as genetic factors were also not been measured.

## Conclusion

The most frequent minor bleeding events of LMWH anticoagulation are hemorrhage and subcutaneous hematoma. This case demonstrated that bowel hematoma despite its low incidence should be considered as an ADR of LMWH therapy, especially among patients who present with gastrointestinal symptoms.

## Data Availability

The raw data supporting the conclusion of this article will be made available by the authors, without undue reservation.
